# Early Detection of Brain Metastases in a Supervised Exercise Program for Patients with Advanced Breast Cancer: A Case Report

**DOI:** 10.1249/MSS.0000000000003213

**Published:** 2023-05-12

**Authors:** MIREIA PELAEZ, MARTIJN M. STUIVER, MARIKE BROEKMAN, KATHRYN H. SCHMITZ, EVA M. ZOPF, DOROTHEA CLAUSS, YVONNE WENGSTRÖM, FRIEDERIKE ROSENBERGER, KAREN STEINDORF, ANDER URRUTICOECHEA, ANNE M. MAY

**Affiliations:** 1 R&D Department, Fundación Onkologikoa, Donostia-San Sebastian, SPAIN; 2 Center for Quality of Life and Division of Psychosocial Research and Epidemiology, Netherlands Cancer Institute, Amsterdam, THE NETHERLANDS; 3 Center of Expertise Urban Vitality, Faculty of Health, Amsterdam University of Applied Sciences, Amsterdam, THE NETHERLANDS; 4 Department of Neurosurgery, Haaglanden Medical Center, The Hague, the Netherlands and Department of Neurosurgery, Leiden University Medical Center, Leiden, THE NETHERLANDS; 5 Department of Medicine, University of Pittsburgh, Pittsburgh, PA; 6 Mary MacKillop Institute for Health Research, Australian Catholic University, Melbourne, Victoria, AUSTRALIA; 7 Cabrini Cancer Institute, Department of Medical Oncology, Cabrini Health, Malvern, Victoria, AUSTRALIA; 8 Department of Molecular and Cellular Sports Medicine, Institute of Cardiovascular Research and Sports Medicine, German Sport University Cologne, Cologne, GERMANY; 9 Theme Cancer, Karolinska University Hospital, Stockholm, SWEDEN; 10 Working Group Exercise Oncology, Department of Medical Oncology, National Center for Tumor Diseases (NCT), Heidelberg University Hospital, Heidelberg, GERMANY; 11 Division of Physical Activity, Prevention and Cancer, German Cancer Research Center (DKFZ) and National Center for Tumor Diseases (NCT) Heidelberg, Heidelberg, GERMANY; 12 Julius Center for Health Sciences and Primary Care, University Medical Center Utrecht, Utrecht, THE NETHERLANDS

**Keywords:** EXERCISE, TRAINING, METASTATIC BREAST CANCER, BRAIN METASTASES, DIAGNOSIS, PREVENTION

## Abstract

**Introduction:**

Around 25% of metastatic breast cancer (mBC) patients develop brain metastases, which vastly affects their overall survival and quality of life. According to the current clinical guidelines, regular magnetic resonance imaging screening is not recommended unless patients have recognized central nervous system–related symptoms.

**Patient Presentation:**

The patient participated in the EFFECT study, a randomized controlled trial aimed to assess the effects of a 9-month structured, individualized and supervised exercise intervention on quality of life, fatigue and other cancer and treatment-related side effects in patients with mBC. She attended the training sessions regularly and was supervised by the same trainer throughout the exercise program. In month 7 of participation, her exercise trainer detected subtle symptoms (e.g., changes in movement pattern, eye movement or balance), which had not been noticed or reported by the patient herself or her family, and which were unlikely to have been detected by the oncologist or other health care providers at that point since symptoms were exercise related. When suspicion of brain metastases was brought to the attention of the oncologist by the exercise trainer, the response was immediate, and led to early detection and treatment of brain metastases.

**Conclusion and clinical implications:**

The brain metastases of this patient were detected earlier due to the recognition of subtle symptoms detected by her exercise trainer and the trust and rapid action by the clinician. The implementation of physical exercise programs for cancer patients requires well-trained professionals who know how to recognize possible alterations in patients and also, good communication between trainers and the medical team to enable the necessary actions to be taken.

The incidence of brain metastases (BM) from breast cancer has increased in recent decades due to improved survival rates for metastatic breast cancer (mBC) and advances in neuroimaging leading to earlier detection (1). Around 25% of mBC patients develop BM (2), which vastly affects their overall survival and quality of life (QoL) (3).

According to the American Society of Clinical Oncology and the European Society for Medical Oncology, regular magnetic resonance imaging (MRI) screening is not recommended unless patients have central nervous system (CNS)-related symptoms (most commonly: headache, nausea, vomiting, focal deficits, or seizures (4)). The downside of this approach, however, is a possible delay in the detection of BM when early symptoms, like subtle changes in balance or movements, go unnoticed, which could reduce the therapeutic options.

Here, we describe a case in which BM of mBC were detected early in a patient who participated in a physical exercise program supervised by a qualified exercise trainer, as part of a randomized controlled trial (RCT). As a result of the close monitoring throughout the intervention, the exercise trainer detected new symptoms, which had not been noticed or reported by the patient herself or her family, and which were unlikely to have been detected by the oncologist at that point. The trainer alerted the oncologist who then acted promptly.

## CASE REPORT

A 75-yr-old woman with a diagnosis of mBC (ER+, PR-, HER2-; Ki-67 10%) participated in the EFFECT study (5). This RCT aims to assess the effects of a structured, individualized, and supervised exercise intervention on QoL, fatigue and other cancer- and treatment-related side effects in patients with mBC, compared with usual care. The exercise program includes two supervised sessions twice a week for 6 months and one supervised session per week from months 6 to 9. Each 1-h session comprises balance (5 min of dynamic, static, and dual tasking exercises), resistance (six loaded exercises at a progressive intensity of 70%–75% or 80%–85% of h1RM) and aerobic training (15 min of moderate-intensity continuous training that progressed to high-intensity interval training on a cycle ergometer). All sessions are supervised by an exercise trainer experienced in training patients with chronic diseases, including cancer (at this site the trainer had more than 15 yr of experience and an academic background in physical activity and sport sciences).

The woman was diagnosed with primary breast cancer in 2014, which had disseminated to the liver in 2016, to the bone in 2017 and to lymph nodes and the kidney in 2020. She had undergone chemotherapy, radiotherapy to the pelvis, endocrine therapy, immunotherapy, and bisphosphonate treatment. At the time of randomization to the intervention group of the EFFECT study (August 2020), she was on her third line of mBC treatment receiving Palbociclib and Fulvestrant. The only known comorbidity was hypercholesterolemia, since 2017, for which she was treated with simvastatin.

The patient attended the training sessions regularly (86%, 43 of 50 sessions). She was supervised by the same exercise trainer throughout the exercise program. In month 7 of the intervention, the patient’s performance declined unexpectedly. During the training session, the trainer recognized some changes and symptoms that were not in line with her previous performance and included failure to complete walking on a line, one leg stand or dual-tasking exercises during the balance component, requiring longer resting intervals or a decrease in intensity (i.e., workload) while performing the high intensity interval protocol during aerobic training. In addition, the patient seemed a little sleepier and her eye movements seemed slower than usual, especially when she was lying down. She also shared a complaint that sometimes her legs did not “listen to her” when she wanted to start walking and that she felt a “bit groggy,” but she did not seem to be overly concerned by it. Initially, the patient attributed these insidious changes to the fluctuations inherent to the disease and its treatments; however, the trainer advised her to talk to her oncologist.

Four weeks later, during a training session, the patient began to make slight superfluous movements with her arms during the bench press exercise. Hence, the trainer encouraged the patient again to speak with her oncologist about these symptoms during the appointment she had that same day, but she did not do so. The patient did not seem to care and was not fully aware of the changes that were happening to her. Hence, these symptoms remained unrecognized by the medical team and the patient’s cancer treatment remained unchanged. Suspecting BM based on the signs that indicated the patient’s neurological functioning was deteriorating, the trainer sought approval from the patient 3 d later, to contact the oncologist and discuss her concerns. The oncologist responded immediately by ordering a brain MRI.

**FIGURE 1:**
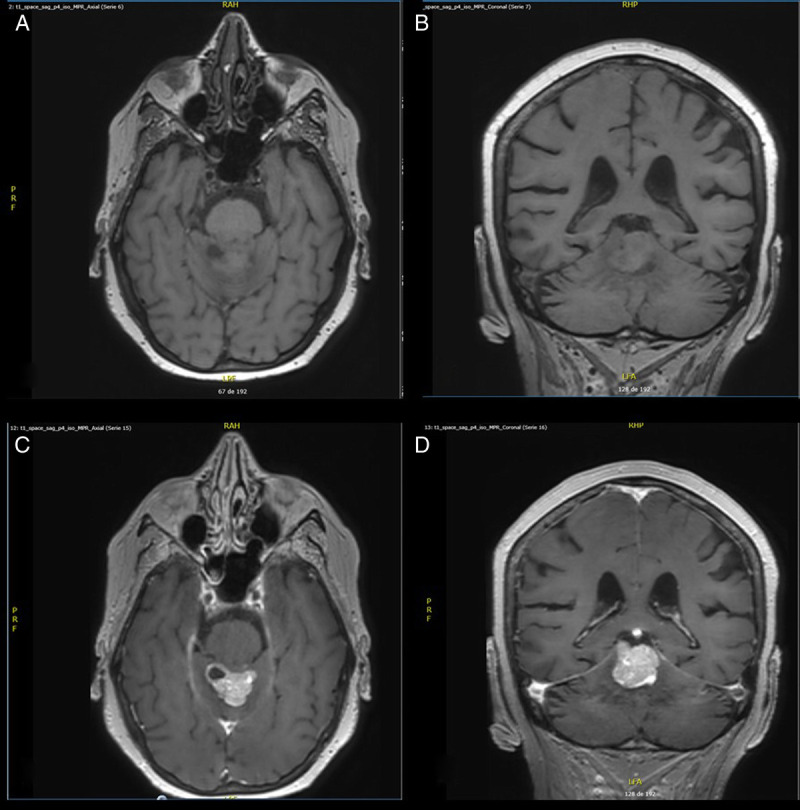
(A) Axial T1 weighted image without gadolinium. (B) Coronal T1 weighted image without gadolinium. (C) Axial T1 weighted image after gadolinium. (D) Coronal T1-weighted image after gadolinium showing an infratentorial lesion suspected for brain metastasis.

Five days after the communication between the trainer and the oncologist, the MRI confirmed BM (Fig. 1). Multiple supratentorial and infratentorial metastases were found, mostly in the cerebellum. Chronic grade one lesions were also observed on the frontal and parietooccipital margins of both semiovale centrum (Fig. 1).

**FIGURE 2:**
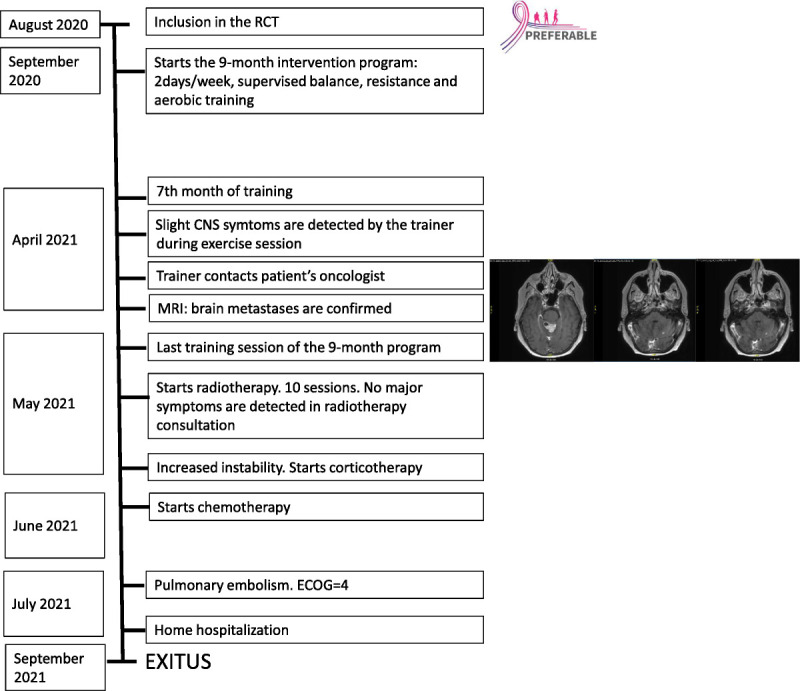
Timeline of the case report. ECOG, Eastern Cooperative Oncology Group.

The new treatment plan included radiotherapy and chemotherapy. At the start of whole brain radiation, 4 wk after the MRI was performed, regular physical examination still showed no obvious signs of CNS involvement. Neither the patient nor her husband believed that there has been a marked deterioration over time. The patient died 5 months after diagnosis of BM. Following the CARE case reports guidelines, a timeline is provided in Figure 2.

## DISCUSSION

This is a case report of a patient with mBC whose BM were detected early as a result of her participation in a supervised exercise program. For this to happen, three aspects were crucial: (i) the close monitoring of the patient by an experienced exercise trainer, (ii) the established communication pathways between the trainer and the medical team, and (iii) the trust in the trainer and hence rapid action by the clinician.

Although the prognosis of BM is currently considered poor, knowledge about cancer dissemination to the brain, prevention and treatments is evolving significantly (6). Improving diagnosis and reducing the time between detection of BM and treatment are key factors that determine prognosis and the patient’s overall survival and QoL (7). Early detection enables early intervention and therefore improves patient outcomes and would also be cost effective, at least in high-risk patients (3). Although we do not know in our specific case if early detection of BM resulted in prolonged survival, early diagnosis and treatment may have prevented convulsions, focal deficits, falls and further loss of QoL, which are the expected consequences of untreated brain metastases (8).

Current guidelines do not recommend MRI screening of the brain unless symptoms are present. The most common symptoms resulting from BM are headache (35%) vomiting (26%), nausea (23%), hemiparesis (22%), visual changes (13%) seizures (12%) (1), and changes in personality. This patient did not experience any of these symptoms, so even close friends or family did not detect warning signs, and neither did the medical team. However, she did show changes in her motor coordination during loaded exercises, in her eye movements and a slight but not easily justified deterioration in her overall performance. Changes such as these are subtle and difficult to notice or assess by a clinician during the short medical appointments. They are, however, likely to be detected by a qualified exercise trainer who supervises the patients while exercising. Since this is a case report, we cannot exclude that also other professionals would have recognized these symptoms. Further, we cannot conclude on how much earlier the BM were detected.

The benefits of physical exercise in cancer patients have been widely corroborated by the literature (9,10). The myriad of both physical and emotional benefits also includes the patients’ perception of feeling safer when exercising under the direct supervision of an exercise trainer, especially if they are properly qualified. In a recent qualitative study, professional supervision was identified as a facilitator for physical exercise participation by patients with mBC, who believed that the constant monitoring of physical functioning might help detect problems that the patient is not yet aware of (11). This case report confirms this.

To conclude, our case report supports the advantages of implementing physical exercise programs that are supervised by qualified professionals as part of routine cancer care for patients in need. This may not only lead to physical and psychological improvements but may also contribute to better patient outcomes through follow-up. We recommend close collaboration between the medical team and exercise professionals because of their unique positioning and skill set to detect subtle deteriorations in health early.

## PRIMARY TAKE AWAY LESSONS OF THIS CASE REPORT

Close monitoring performed by qualified exercise trainers during supervised exercise sessions can lead to the detection of subtle but significant signs of disease progression that may not be recognized yet by clinicians, patients or their family. Early detection of disease progression can make a significant difference to the patient’s QoL and life expectancy.

We recommend that changes in movement patterns that become uncoordinated or less forceful, gait disturbance, a deterioration in balance, slower or clumsier eye movements, changes in mental state or behavior (more angry, uninhibited, disconnected, sleepy, etc.), or any unexpected performance decline recognized by a trainer should be brought to the patient’s attention.
